# Non-Decaying postsynaptics potentials and delayed spikes in hippocampal pyramidal neurons generated by a zero slope conductance created by the persistent Na^+^ current

**DOI:** 10.1080/19336950.2018.1433940

**Published:** 2018-02-16

**Authors:** Cesar C. Ceballos, Rodrigo F. O. Pena, Antônio C. Roque, Ricardo M. Leão

**Affiliations:** a Department of Physiology, School of Medicine of Ribeirão Preto, University of São Paulo, Ribeirão Preto, SP, Brazil; b Department of Physics, School of Philosophy, Sciences and Letters, University of São Paulo, Ribeirão Preto, SP, Brazil

**Keywords:** Zero slope conductance, infinite membrane time constant, persistent sodium current, non-decaying postsynaptic potential, spike latency

## Abstract

The negative slope conductance created by the persistent sodium current (I_NaP_) prolongs the decay phase of excitatory postsynaptic potentials (EPSPs). In a recent study, we demonstrated that this effect was due to an increase of the membrane time constant. When the negative slope conductance opposes completely the positive slope conductances of the other currents it creates a zero slope conductance region. In this region the membrane time constant is infinite and the decay phase of the EPSPs is virtually absent. Here we show that non-decaying EPSPs are present in CA1 hippocampal pyramidal cells in the zero slope conductance region, in the suprathreshold range of membrane potential. Na^+^ channel block with tetrodotoxin abolishes the non-decaying EPSPs. Interestingly, the non-decaying EPSPs are observed only in response to artificial excitatory postsynaptic currents (aEPSCs) of small amplitude, and not in response to aEPSCs of big amplitude. We also observed concomitantly delayed spikes with long latencies and high variability only in response to small amplitude aEPSCs. Our results showed that in CA1 pyramidal neurons I_NaP_ creates non-decaying EPSPs and delayed spikes in the subthreshold range of membrane potentials, which could potentiate synaptic integration of synaptic potentials coming from distal regions of the dendritic tree.

## Introduction

Excitatory postsynaptic potentials (EPSPs) are shaped by subthreshold voltage dependent currents [1]. The persistent sodium current (I_NaP_) is a subthreshold non-inactivating voltage dependent current with a similar voltage-dependence of classical transient sodium current, but with more negative activation potentials. I_NaP_ has a non-monotonic I-V relationship displaying a negative slope conductance (dI/dV) at its activation phase that increases the input resistance and membrane time constant (τ_m_) [1-3]. Moreover, I_NaP_ is generated by the same Na_V_ subunits that produce transient Na^+^ current, and depends on the interaction of the Na_V_ alpha subunit with regulatory beta subunits [4,5]. Several alpha subunits can produce I_NaP_. For instance, the Na_V_ 1.6 in CA1 pyramidal cells and the Na_V_ 1.7 in neurons of the hypothalamic arcuate nucleus [6,7]. Thus, I_NaP_ is not restrict to a specific subunit and can be expressed in several neuronal types.

In a recent combined experimental and computer simulation study [2] we showed that the negative slope conductance of tetrodotoxin-sensitive I_NaP_ is the main responsible for the prolongation of the decay phase of EPSPs in hippocampal pyramidal neurons in the voltage region near spike threshold. The negative slope conductance of I_NaP_ decreases the positive slope conductance of leak currents, leading to a net increase of the input resistance and consequently to an increase of τ_m_. We showed that the negative slope conductance of I_NaP_ increases with depolarization, resulting in gradual increase of τ_m_ and consequently increase of prolongation of the EPSPs. Interestingly, if the membrane potential is further depolarized, a region of zero slope conductance might emerge (i.e. a point where the I–V slope is flat). Theoretical and experimental studies have demonstrated that regions with zero slope conductance imply an infinite τ_m_ [8] which could produce non-decaying EPSPs. Remarkably, in some neurons, called perfect integrators, the near-threshold EPSPs are exclusively non-decaying [6,9,10]. In addition, delayed spikes evoked by non-decaying EPSPs have been observed in these neurons, within the zero slope conductance region [6,9,10].

Are the non-decaying EPSPs exclusively found in perfect integrators neurons? To the best of our knowledge, there is no study on non-decaying subthreshold EPSPs in hippocampal CA1 pyramidal neurons, although a region of zero slope conductance has been observed in these neurons, in voltage clamp recordings using slow ramps [2,3]. Thus, we decided to investigate if CA1 pyramidal neurons display non-decaying EPSPs at its zero slope conductance region created by I_NaP_. For such purpose, we applied transient voltage changes by artificial excitatory postsynaptic currents in different potentials [2]. We found that the I_NaP_ created longer EPSPs, several with a non-decaying profile. This effect was observed only above activity threshold, and only in response to small depolarizations. We also found delayed spikes produced by only small suprathreshold depolarizations. We conclude that the zero slope conductance created by I_NaP_ is the main mechanism underlying the generation of non-decaying EPSPs and delayed spikes in hippocampal pyramidal neurons.

## Results

### CA1 pyramidal cells have a suprathreshold zero slope conductance region

**Figure 1.**
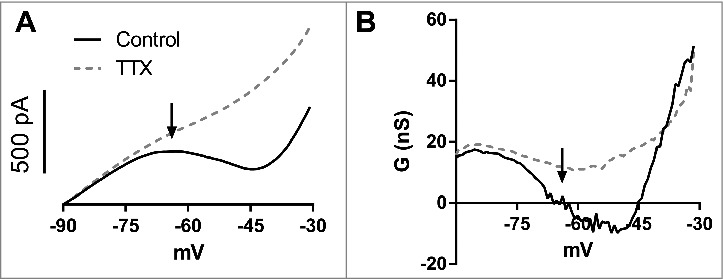
A. Mean slow ramps (15 mV/s) recorded in voltage-clamp from -90 mV to -30 mV in control aCSF and after TTX (n = 10). B. Slope conductance (dI/dV) in control aCSF and after TTX obtained from curves in A. Note that the zero slope conductance region (arrows) is near -65 mV and corresponds to an unstable equilibrium.

When we performed slow ramps (15 mV/s) in voltage clamp, we observed a narrow region of zero slope conductance between -70 and -60 mV (Fig. 1A,B). This region represents an unstable equilibrium of both inward and outward currents [3]. After this, at more positive potentials, there is a region of negative slope conductance followed by other zero slope conductance point and finally a positive slope conductance. The first zero slope conductance region is above the activity threshold [11] of these neurons (activity threshold -62.9 ± 1.2 mV,n = 6). The activity threshold is defined as the potential, below the action potential threshold, in which a neuron starts to present spontaneous firing, and is caused by the activation of subthreshold inward currents as the I_NaP_ [11]. From now on we will refer to events above the activity threshold as suprathreshold, and below the activity threshold, as subthreshold.

After inhibiting voltage-dependent sodium channels with tetrodotoxin (TTX), only positive slope conductance remains, suggesting that the negative and zero slope conductance regions are mainly generated by the negative slope conductance of I_NaP_.

### Suprathreshold non-decaying EPSPs due to the zero slope conductance

It is expected, then, that in a region of zero slope conductance, τ_m_ is almost infinite resulting in non-decaying EPSPs and in spikes with long latencies [6,9,10]. In order to determine if in hippocampal CA1 neurons the zero slope conductance largely mediated by I_NaP_ can produce non-decaying EPSPs and long-latency spikes, we produced either small or big artificial EPSPs by injecting triangular fast depolarizations (artificial excitatory postsynaptic currents, aEPSC, 100 or 200 pA, respectively) at potentials below and above the activity threshold. We recorded the membrane responses before and after the application of TTX to inhibit I_NaP_.

**Figure 2.**
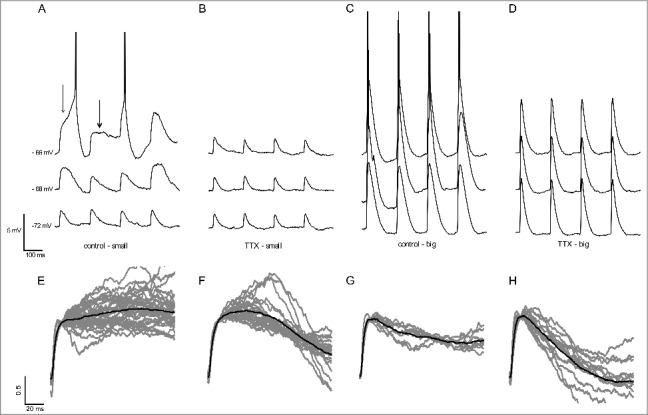
A. Representative traces of events evoked by small aEPSCs for suprathreshold (-66 mV) and subthreshold membrane potentials (-68 mV and -72 mV) in the control aCSF and after TTX (B). The thin arrow shows a delayed spike and the thick arrow shows a non-decaying EPSP. C. Representative traces of events evoked by big aEPSCs for suprathreshold and subthreshold membrane potentials in the control aCSF and after TTX (D). Only fast spikes and decaying EPSPs are observed. E-H. Classification of the EPSPs evoked by small aEPSCs at suprathreshold potentials. E. Non-decaying EPSPs (n = 36). F. Decaying EPSPs with pronounced initial amplification (n = 24). G. EPSPs with slow monotonic decay (n = 8). H. EPSPs with fast monotonic decay (n = 14). The dark trace is the average of the individual EPSPs (gray).

We observed that in potentials near the resting membrane potential, the small EPSPs had the shape similar of the aEPSCs, suggesting little activation of active conductances, as I_NaP_, at these potentials (Figure 2A). At potentials near the activity threshold we observed broader EPSPs (Figure 2A), and in potentials above the activity threshold we observed non-decaying EPSPs and delayed spikes evoked by the EPSPs (Fig. 2A). But, we still observed decaying EPSPs and short latency spikes (Fig. 2A). After TTX the non-decaying EPSPs and the longer EPSPs were abolished, showing that these types of EPSPs were mainly caused by I_NaP_ activation (Fig. 2B). Thus, we conclude that the broader EPSPs are mainly caused by the negative conductance window of I_NaP_ and the non-decaying EPSPs are caused by the zero slope conductance region.

Interestingly, bigger EPSPs were not able to produce neither non-decaying EPSPs nor delayed spikes (Figure 2C). However, this was not due to a lack of I_NaP_ influence, since we observed that after application of TTX, the EPSPs showed a decrease of their size and duration (Figure 2C, 2D). Because the absence of delayed spikes evoked by bigger EPSPs was concomitant with the absence of non-decaying EPSPs, we suggest that both are caused by the same underlying mechanism, the zero slope conductance region.

We observed non-decaying EPSPs and delayed spikes evoked by small EPSPs, in potentials above the activity threshold, in all neurons recorded (n = 15). In order to classify the non-decaying and decaying suprathreshold EPSPs, we used a decay index (DI) defined as the ratio between two amplitudes in the same EPSP at different times (DI = amp_t1_/amp_t2_). We compared the EPSP amplitude just after 10 ms (initial rise phase) with the amplitudes after 50 ms (middle) and 100 ms (late) after the rise phase. We estimated three DI, DI_1_ = middle/initial, DI_2_ = late/initial and DI_3_ = middle/late. We set an arbitrary, but intuitive, cutoff of all indexes: above 0.8 for the 3 DIs for non-decaying EPSPs and below this value for decaying EPSPs. Figure 2E shows all the traces for non-decaying EPSPs in gray and the mean trace in black, and Figures 2F-H the traces of the decaying EPSPs (Fig. 2F, 2G and 2H). We observed a high heterogeneity in the shape of decaying EPSPs and for this reason we classified them in three subclasses: EPSPs with pronounced initial amplification (DI_1_ > 0.8 and DI_2_< 0.8; Fig. 2F), with slow monotonic decay (0.5 < DI_1_< 0.8 and 0.5 < DI_2_< 0.8; Fig. 2G) and fast monotonic decay (DI_1 _< 0.5 and DI_2_ < 0.5; Fig. 2H).

At all membrane potentials, we detected, in total, 39 non-decaying EPSPs, being 36 in the supratheshold potentials and 3 in potentials below the activity threshold, suggesting that, in the subthtreshold region, the non-decaying EPSPs are rare. Furthermore, we detected 46 decaying EPSPs in the suprathreshold potentials. Thus, 44% of the EPSPs in the suprathreshold potentials were non-decaying. In addition, we also estimated the probability to evoke a spike or a non-decaying EPSP at suprathreshold potentials. At the lowest membrane potential, in which at least one out of four EPSPs evoked one spike, we had 15% probability of having a non-decaying EPSP and 54% probability of having a spike. Further depolarization of the membrane potential decreased the probability to evoke a non-decaying EPSP (10%) and increases the probability to evoke a spike (69%). Our results show that non-decaying EPSPs are mainly present in the lower suprathreshold voltage range and are equally common as decaying EPSPs. However as we depolarize the neuron further we decrease the detection of non-decaying EPSPs, and increase the detection of spikes, which could be triggered by both decaying and non-decaying EPSPs. Nevertheless because the delayed spikes are only seen with the small EPSPs, which produce the non-decaying EPSPs, we believe that most of the delayed spikes are actually non-decaying EPSPs that generate spikes [9]. So the number of non-decaying EPSPs could be underestimated.

### The zero slope conductance generates delayed spikes evoked by small EPSPs

**Figure 3.**
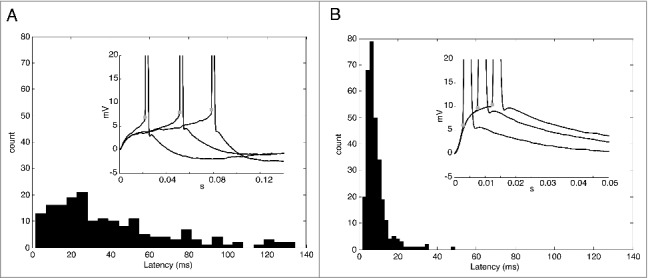
A. Histogram of latencies for delayed spikes evoked by small EPSCs. Inset. Representative traces of delayed spikes. The values are normalized by the baseline. Stars show the spike threshold (dV/dt = 10 mV/ms). B. Histogram of latencies for delayed spikes evoked by big EPSCs. Inset. Representative traces of delayed spikes. The values are normalized by the baseline. Stars show the spike threshold (dV/dt = 10 mV/ms).

We believe that most of the delayed spikes are actually non-decaying EPSPs that generate spikes [6,9,10]. Thus, we also characterized the presence of delayed spikes by means of the latency calculated as the time between the EPSP onset and the spike threshold (the action potential threshold, see methods). We performed this analysis in spikes evoked by small and big EPSPs (Fig. 3A and 3B). Interestingly, we found a broad range of the latencies for the spikes evoked by small EPSPs up to 150 ms (Fig. 3C), (which was our maximum temporal window). On the other hand, the range of the latencies of the spikes evoked by big EPSPs was narrower and restricted to values below 50 ms (Fig. 3D). The mean values were 40 ± 2.3 ms for small EPSPs and 8.8 ± 0.3 ms for big EPSPs. The presence of delayed spikes, probably caused by the zero slope conductance region, can increase the probability of a small EPSP generate a spike, but, on the other hand, with a poor spike time precision. Interestingly, we found a positive correlation of spike threshold and latency (R^2^ = 0.44; *p* < 0.0001; not shown).

## Discussion

This study follows our previous study [2] where we demonstrated that the negative slope conductance of I_NaP_ is the main responsible for the increase in membrane time constant in near threshold potentials, prolonging EPSPs at this potential range. Here we provide evidences that a region of zero slope conductance is created by the negative slope conductance of I_NaP_ prolonging the membrane time constant to an almost infinite value. This allows that non-decaying EPSPs and delayed spikes emerge [3,12]. In the CA1 pyramidal cells, we found that this zero slope conductance region is located in the range of the membrane potentials just above the activity threshold, in line with a previous work [3] reporting an analysis similar to ours, in CA1 pyramidal cells of the effects of I_NaP_ at the same voltage range as we used (near-threshold, -60 mV). As we did, they observed that I_NaP_ produces an increase in membrane time constant in the near-threshold regions, resulting in delayed spikes observed as small plateaus preceding spikes.

We found that in potentials near the activity threshold, the EPSPs become prolonged, in accordance to the effect of the negative conductance created by I_NaP_, on the membrane time constant [2,3]. Interestingly in potentials above the activity threshold, we observed non-decaying EPSPs, suggestive of the effect of the zero slope conductance region created during the activation of I_NaP_. Because application of TTX abolished both the non-decaying EPSPs and the prolonged EPSPs we conclude that they are largely mediated by I_NaP_. Nevertheless, we cannot exclude some partial contribution of other negative conductances (e.g. Ca or NMDA) that also prolong the EPSPs [1] and that are activated in voltages nearby the zero slope conductance region.

Interestingly, only small but not big EPSCs produced non-decaying EPSPs and delayed spikes. This difference is not caused by differential activation of the persistent and transient sodium current, since both transient and persistent sodium currents are largely recruited by both rapid (like EPSPs) or slow voltage fluctuations [3,13]. This highlighted the dependency of the non-decaying EPSPs on the slow depolarization rate of the membrane (dV/dt) of the EPSPs created by small aEPSCs. We believe that during slower depolarizations, as produced by the small aEPSCs, there is a relative inactivation of a fraction of the voltage-dependent sodium channels, delaying the firing of action potentials. In accordance we found a correlation of the latency with the spike threshold, suggesting that more sodium channels are inactivated during the slow depolarizations produced by the small EPSPs. Thus, large EPSPs are able to fire spikes with low latencies due to its large rate of depolarization that allows the fast activation of the transient sodium current without significant inactivation [3,13-15] and, by this, preventing the formation of the zero-conductance region necessary for non-decaying EPSPs and delayed spikes. In addition, concomitant activation of low-threshold voltage-activated K^+^ currents by big but not small EPSPs can curtail the delayed spikes [16]. Interestingly we found that TTX shortened the big EPSPs, showing that the negative conductance of I_NaP_ is effective in prolonging these EPSPs, but the fast rise of depolarization impairs the formation of non-decaying EPSPs and delayed spikes.

Our results on the generation of delayed spikes are in agreement with previous studies in cortical and hippocampal pyramidal neurons [3,17-19]. In these studies, delayed spikes evoked by EPSPs were observed only when the initial membrane potential was near threshold. On the other hand, big EPSPs evoked from hyperpolarized membrane potentials fired spikes with low latencies and small variability. For instance, in CA1 pyramidal cells, the latency of spike initiation becomes more variable as the membrane potential approaches the spike threshold, but for further depolarization, the action potentials are reliably initiated at a short and constant latency [20]. This is in agreement with our hypotheses, since at more depolarized membrane potentials, the zero slope conductance disappears and a region of negative slope conductance is dominant. Consistent with this hypothesis, large latency variability has been observed in neurons with zero slope conductance that displays non-decaying EPSPs [6,9,10]. In agreement with the participation of I_NaP_ on the generation of delayed spikes, cancelling endogenous I_NaP_ in CA1 pyramidal cells decreases the values and variability of spike latencies [21] and knocking-out Na_V_1.7, which produces I_NaP_ in AGRP neurons, resulted in a decrease of spike latencies [6]. Furthermore, it has been shown that currents with positive slope conductance, such as K^+^ currents and I_h_ decrease the variability of the spike latencies [15,22,23], meanwhile currents with negative slope conductances such as Ca^++^ or NMDA currents increase the variability of the spike latencies [1,10,24].

But how could non-decaying EPSPs, decaying EPSPs and delayed spikes be generated in the same voltage region? We propose that this can be explained by understanding that the zero slope conductance region corresponds to an unstable equilibrium of opposing conductances. According with this, small displacements moving out from the zero slope conductance region drives the neuron membrane potential to a positive or negative slope conductance region. Thus, non-decaying EPSPs emerge when the voltage enters this small region of the zero slope conductance and the net current drops to almost zero. For this, the contribution of inward and outward currents must be cancelled. If the inward current is bigger than the outward current, then a delayed spike emerges. In contrast, if the inward current is smaller than the outward current, then a decaying EPSP emerges. If the transient sodium current is rapidly activated with little inactivation, then a spike without delay is fired. In agreement with this, a computational model showed that differences in balance between inward/outward currents determine if a delayed spike, non-decaying EPSPs or classical decaying EPSPs will evoke from EPSPs [6]. In this model, the authors demonstrated that EPSP prolongation occurs in a narrow band of I_NaP_ and voltage-gated potassium conductances.

So, the negative conductance of I_NaP_ not only prolong near-threshold EPSPs, increasing the temporal integration window and enhancing rate coding of CA1 pyramidal neurons [25], but supports the generation of non-decaying EPSPs and delayed spikes in response to small EPSPs in the active range of the neurons. This boosts the effect of small and slow EPSPs, probably generated in the distal dendrites, in generating action potentials, but with a lower temporal precision. Thus, we conclude that a region of zero slope conductance, created by I_NaP_, endows hippocampal CA1 pyramidal neurons with a longer temporal integration window of small synaptic inputs near spike threshold, which could potentiate the integration of the information conveyed by dendritic distal inputs, overcoming, at least partially, the dendritic filtering of these signals.

Our results suggest that the effects of I_NaP_ on EPSP summation and the temporal precision of EPSP-spike coupling oppose those exerted by I_h_. While I_NaP_ widens the temporal window for EPSP summation and increases the variability of spike latencies, I_h_ produces opposite effects [22,26]. These effects are the result of the opposite effects of I_NaP_ and I_h_ on the membrane time constant [3]. Interestingly, I_NaP_ and I_h_ are segregated by the range of the voltage activation [3] and the location in different neuronal compartments. While I_NaP_ is mostly expressed in the soma and axonal initial segment and activated at depolarized membrane potentials [3,17,20], I_h_ is mostly expressed in the dendrites and activated at hyperpolarized membrane potentials [3,27]. This suggests that CA1 pyramidal cells act like coincident detectors for small EPSPs at hyperpolarized membrane potentials in dendrites but they behave like integrators at depolarized membrane potentials for small EPSPs in the soma.

To our knowledge, this is the first study showing non-decaying EPSPs in a neuron that is not a perfect integrator as has been reported elsewhere [6,9,10]. This suggests that non-decaying EPSPs and delayed spikes are likely to exist in all types of neurons in the region of zero slope conductance. The lack of more studies showing non-decaying EPSPs could be due to that they are more likely to exist in the suprathreshold region where spontaneous spikes are also present, a region not usually studied. We conclude that non-decaying EPSPs could be a general feature of neurons expressing I_NaP_, but that its existence might be restricted to a narrow region of the membrane potentials in which a region of zero slope conductance exists.

## Methods

All the methods used for the hippocampal slices preparation and patch clamp recordings have been described previously [2]. The animal experimental procedures were approved by the Ethics Committee on Animal Experimentation (CEUA) of the School of Medicine of Ribeirão Preto. Briefly, male Wistar rats (P18 to P22) were anesthetized with isoflurane, decapitated and their brains removed. Coronal slices (300 µm thick) containing the dorsal hippocampus were obtained with a vibratome. Whole-cell patch-clamp recordings were performed in CA1 pyramidal neurons visualized with a microscope equipped with DIC-IR optics using a 40x water immersion objective. Recordings were obtained with borosilicate microelectrodes presenting a resistance between 4–6 MΩ when filled with a potassium gluconate based internal solution. In voltage clamp, series resistance was compensated at 80%. All the recordings were done in the presence of the GABA_A_ antagonist picrotoxin (PTX, 20 µM). The liquid junction potential (10 mV) was subtracted off-line.

To measure the slope conductance, voltage ramps were applied from −90 mV to −30 mV for 4 seconds before and after TTX (100 nM). Spike threshold was measured from phase plots of the action potentials as the voltage in which dV/dt = 10 mV/ms [11]. V-I curves in current-clamp were obtained by injecting 1 s depolarizing current pulses with +20 pA steps from 0 pA to +400 pA. In order to determine the effect of the I_NaP_ on the amplitude and duration of the EPSP, four artificial EPSCs (aEPSCs) were injected at the end of the pulse, with a separation of 150 ms, while the membrane potential was changed. aEPSCs were built using two consecutive current ramps (2 ms rising and 5 ms decay). Small EPSCs had amplitude of 100 pA and big EPSCs amplitude of 200 pA. This was set to obtain an aEPSP of ∼ 2 mV or 5 mV at -90 mV, respectively. Data were analyzed using programs written in Igor Pro and Matlab. Data are presented as mean ± SEM.

## Abbreviations

*EPSP*: Excitatory postsynaptic potential
*I_NaP_*: persistent sodium current.
